# Insights Into the Bifunctional Aphidicolan-16-ß-ol Synthase Through Rapid Biomolecular Modeling Approaches

**DOI:** 10.3389/fchem.2018.00101

**Published:** 2018-04-10

**Authors:** Max Hirte, Nicolas Meese, Michael Mertz, Monika Fuchs, Thomas B. Brück

**Affiliations:** Werner Siemens Chair of Synthetic Biotechnology, Department of Chemistry, Technical University of Munich, Munich, Germany

**Keywords:** homology modeling, aphidicolin, diterpene, diterpene synthase, homology model refinement

## Abstract

Diterpene synthases catalyze complex, multi-step C-C coupling reactions thereby converting the universal, aliphatic precursor geranylgeranyl diphosphate into diverse olefinic macrocylces that form the basis for the structural diversity of the diterpene natural product family. Since catalytically relevant crystal structures of diterpene synthases are scarce, homology based biomolecular modeling techniques offer an alternative route to study the enzyme's reaction mechanism. However, precise identification of catalytically relevant amino acids is challenging since these models require careful preparation and refinement techniques prior to substrate docking studies. Targeted amino acid substitutions in this protein class can initiate premature quenching of the carbocation centered reaction cascade. The structural characterization of those alternative cyclization products allows for elucidation of the cyclization reaction cascade and provides a new source for complex macrocyclic synthons. In this study, new insights into structure and function of the fungal, bifunctional Aphidicolan-16-ß-ol synthase were achieved using a simplified biomolecular modeling strategy. The applied refinement methodologies could rapidly generate a reliable protein-ligand complex, which provides for an accurate *in silico* identification of catalytically relevant amino acids. Guided by our modeling data, ACS mutations lead to the identification of the catalytically relevant ACS amino acid network I626, T657, Y658, A786, F789, and Y923. Moreover, the ACS amino acid substitutions Y658L and D661A resulted in a premature termination of the cyclization reaction cascade *en-route* from syn-copalyl diphosphate to Aphidicolan-16-ß-ol. Both ACS mutants generated the diterpene macrocycle syn-copalol and a minor, non-hydroxylated labdane related diterpene, respectively. Our biomolecular modeling and mutational studies suggest that the ACS substrate cyclization occurs in a spatially restricted location of the enzyme's active site and that the geranylgeranyl diphosphate derived pyrophosphate moiety remains in the ACS active site thereby directing the cyclization process. Our cumulative data confirm that amino acids constituting the G-loop of diterpene synthases are involved in the open to the closed, catalytically active enzyme conformation. This study demonstrates that a simple and rapid biomolecular modeling procedure can predict catalytically relevant amino acids. The approach reduces computational and experimental screening efforts for diterpene synthase structure-function analyses.

## Introduction

With more than 50,000 different molecules known to date terpenes are the greatest natural occurring product family found in organisms from bacteria to fungi, mammals, and plants. They are all derived from the isoprene units' dimethylallyl diphosphate and isopentenyl diphosphate. Condensation reactions of this molecules lead to the formation of different length phosphorylated linear terpenes, serving as substrate for terpene synthases. This enzyme family carry out highly stereo complex C-C coupling reactions, resulting in structurally complex macrocycles that contribute to the structural and functional diversity of terpenes (Christianson, 2017). Diterpenes are derived from the linear aliphatic precursor geranylgeranyl diphosphate (GGDP) being cyclized by diterpene synthases. More specifically, diterpene synthases are classified into class I and class II enzymes based on the structural presence of the conserved motifs DDXD or DDXXD/E and NSE/DTE, respectively. While class II reactions perform a protonation initiated cyclization reaction to generate phosphorylated bicyclic structures, class I reactions are initiated by hydrolyses of the GGDP pyrophosphate moiety that is coordinated by a Mg^2+^-triad thereby generating mono- or poly-cyclic structures.

**Figure 1:**
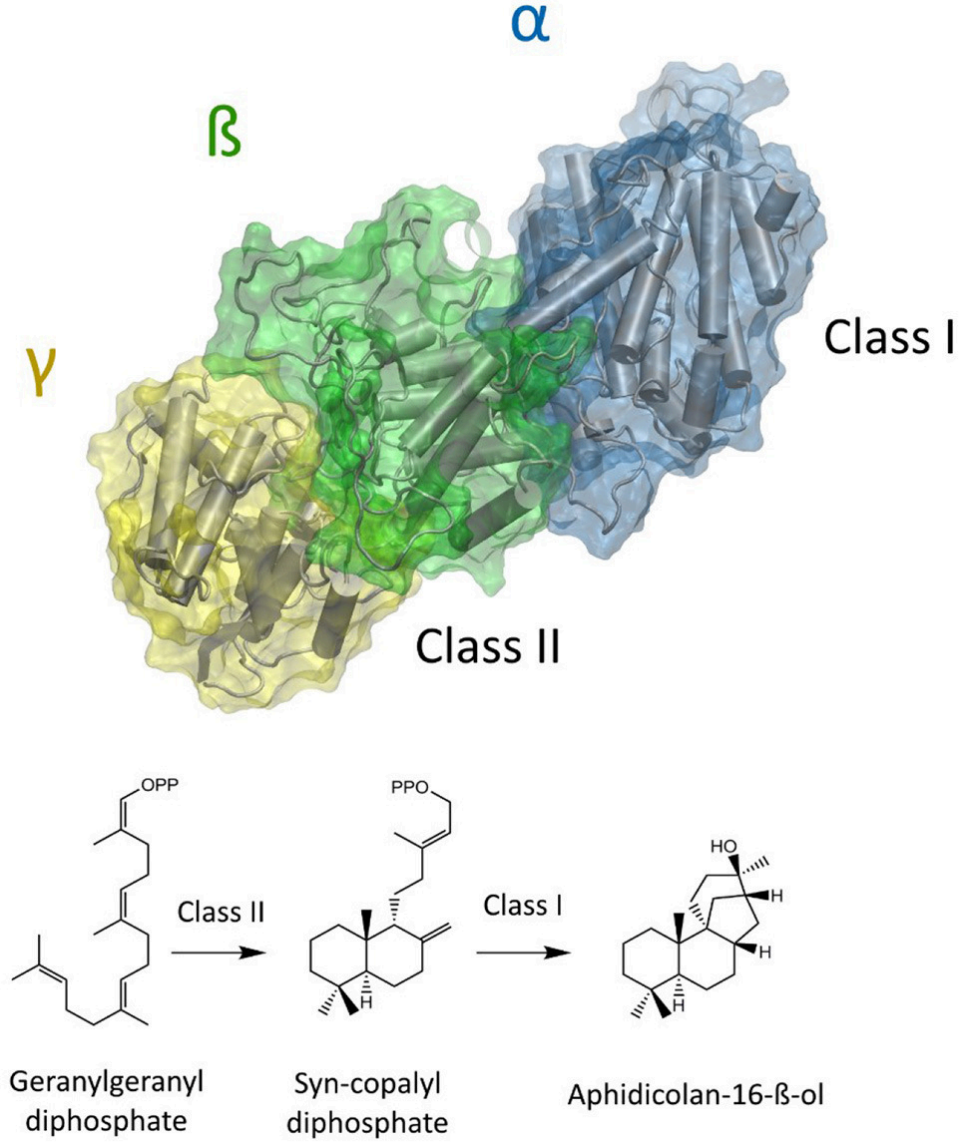
Model of a bifunctional diterpene synthase. In the case of ACS GGDP is initially converted to syn-CDP in the class II active site (located between ß and γ domain). Syn-CDP is further cyclizied to AD in class I active site (α-domain).

The natural product Aphidicolin, initially isolated from the fungus *Cephalosporium aphidicola*, is a hydroxylated, tetracyclic diterpenoid that exhibits a broad range of biological activities and applications (Brundret et al., 1972; Dalziel et al., 1973). More specifically, it is a potent inhibitor of the eukaryotic DNA α-polymerase with a commercial application as a cell synchronization agent. The compound is in pharmaceutical development due anti-tumor, anti-viral, and anti-leishmanial activity (Ikegami et al., 1978; Pedrali-Noy et al., 1980; Kayser et al., 2001; Edwards et al., 2013; Starczewska et al., 2016). Recently, other organisms including the fungus *Nigrospora sphaerica* and the pathogenic fungus *Phoma betae* have been identified as natural Aphidicolin producers. Current data suggests that Aphidicolin biosynthesis is exclusive to fungal metabolism and that natural sources for Aphidicolin are limited (Starratt and Loschiavo, 1974; Fujii et al., 2011; Lopes and Pupo, 2011). Nevertheless, elucidation of the responsible Aphidicolin biosynthetic gene cluster in *P. betae* allowed for the identification of a bifunctional diterpene synthase that contains both a functional class I and class II domain (Oikawa et al., 2001). The Aphidicolan-16-ß-ol synthase (ACS) generates the stereo-chemically demanding Aphidicolan-16-ß-ol (AD)—core structure of Aphidicolin—structure via a two-step reaction as depicted in Figure 1 (Oikawa et al., 2002).

**Figure 2:**
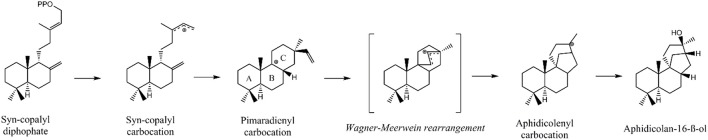
Proposed cyclization mechanism of the ACS class I reaction (Oikawa et al., 2002).

Initially, GGDP is rearranged in the class II active site cleft by protonation to the bicyclic syn-copalyl diphosphate (syn-CDP). Subsequently, syn-CDP is elaborated to AD in the class I active site (Adams and Bu'Lock, 1975; Oikawa et al., 2002). As depicted in Figure 2 the cyclization mechanism in the class I active site, initiated by the hydrolysis of the pyrophosphate group, results in 8-ß-pimaradienyl carbocation formation. A subsequent attack of the vinyl group, bridging the C ring, directly undergoes a Wagner-Meerwein rearrangement and results in the formation of the aphidicolenyl carbocation. Eventually, this cation is quenched by water thereby generating AD.

Terpene cyclization mechanisms are conventionally elucidated by radio labeling of protons and carbons (Dickschat, 2017). This substrate specific labeling provides for identification of unusual hydride shifts and rearrangements. Alternatively, the enzyme's cyclization mechanisms can be probed by altering amino acids, trying to terminate the reaction cascade at a specific transition state (Morrone et al., 2008; Janke et al., 2014; Schrepfer et al., 2016; Jia et al., 2017). Therefore, random mutagenesis can be performed but the screening effort for this methodology is elaborate without an efficient high throughput screening options (Lauchli et al., 2013). Biomolecular modeling allows for the rational identification and *in silico* modulation of amino acid networks that are involved in complex reaction cascades (Pemberton et al., 2015; Schrepfer et al., 2016; Christianson, 2017; Escorcia et al., 2018). This methodology provides for a knowledge based approach of enzyme mutagenesis and screening. Nevertheless, a particular challenge for this strategy is based on the missing structural information for most terpene synthases. However, as their structural elements and domains are highly conserved (Christianson, 2017), homology modeling is a potential route to identify catalytically relevant amino acids despite the low primary sequence identities in this enzyme family (Xu and Li, 2003). Unfortunately, most available crystal structures of terpene synthase are deposited in the open apo-enzyme configuration that is catalytically inactive. This open enzyme conformation presents an additional obstacle when catalytically relevant amino acids have to be identified *in silico*. At present, only two diterpene synthase structures have been reported in the closed, catalytically active form (Liu et al., 2014; Serrano-Posada et al., 2015). Therefore, automated homology modeling approaches will almost always result in catalytically non-relevant open enzyme configuration. Moreover, while prediction tools can place large cofactors (i.e., FAD, NADH, Heme) correctly in the apo-protein framework, ligand-metal interactions are difficult to predict because of the multiple coordination geometries and the lack of sufficiently accurate force field parameters (Khandelwal et al., 2005). Hence, structure function predictions that depend on the interplay between the amino acids of the protein framework with small metal ions cannot be conducted solely by application of automated software tools. In this context, a rational combination of structural information by superposition and extraction of cofactors is performed to prepare the protein structure for docking studies. Nevertheless, this approach often neglects reliable positioning of the cofactor coordinating amino acids. Additionally, falsely predicted positioning of amino acid side chains in the active site cleft can lead to invalid interpretation of a homology model based protein-ligand complex. To improve this situation, this study elucidated rapid and simple methodologies to refine diterpene homology models for docking studies thereby allowing for reliable structure-function predictions. In this context, an ACS class I homology model of the α-domain was predicted from the primary sequence. Subsequently, these models were compared to catalytically relevant closed terpene synthases structures. The location of metals was refined and fitted against specifically selected structural templates and multiple docking studies were carried out and validated. Our *in silico* results were experimentally evaluated by ACS mutagenesis studies. This lead to an identification of essential amino acid residue sidechains that are necessary for retaining the enzymes activity. Additionally, we detected amino acid substitutions that abort the catalytic reaction cascade *en- route* from syn-CDP to AD. Structural analyses and elucidation of these compounds revealed the formation of syn-copalol and a labdane related, non-hydroxylated diterpene by the ACS mutants Y658L and D661A. Our approach of a protein homology model based structure function analysis can be easily adapted for other terpene synthases. This methodology allows for rapid and simple analysis of the catalytically relevant amino acid network that help studying complex reaction cascades and developing new biocatalysts.

## Materials and methods

### Materials and chemicals

All genes used were synthesized by Life technologies GmbH and the codon usage was optimized for *E. coli* if not stated otherwise. Primers were obtained from Eurofins Genomics GmbH. Strains and plasmids were obtained from Merck KGaA. All chemicals used were obtained at highest purity from Roth chemicals or Applichem GmbH. Enzymes were purchased from Thermo Fisher Scientific.

### Software and web-tools

RaptorX was applied for homology modeling studies (http://raptorx.uchicago.edu; Källberg et al., 2012). The initial predicted structure was analyzed and further modified in the environment of UCSF Chimera software package (Pettersen et al., 2004; http://www.cgl.ucsf.edu/chimera). Comparative modeling by spatial restraints was performed by MODELLER (Eswar et al., 2006), and all substrate docking studies performed by AutoDock Vina (Trott and Olson, 2010; http://vina.scripps.edu). Chemical structures were drawn by PerkinElmer ChemBioDraw Ultra (http://www.cambridgesoft.com). For ligand preparation the Avogadro (Hanwell et al., 2012; https://avogadro.cc/) software package was used. A syn-CDP toppar stream file was generated by CHARMM General Force Field program version 1.0.0 for use with CGenFF version 3.0.1 (https://cgenff.paramchem.org; Vanommeslaeghe et al., 2010, 2012; Vanommeslaeghe and MacKerell, 2012). Two ns molecular dynamic studies of the docked ACS model B in a water sphere have been performed under CHARMM general force field by NAMD (Phillips et al., 2005; http://www.ks.uiuc.edu/Research/namd/). NAMD was developed by the Theoretical and Computational Biophysics Group in the Beckman Institute for Advanced Science and Technology at the University of Illinois at Urbana-Champaign. For high resolution pictures the protein was prepared by Visual Molecular Dynamics (http://www.ks.uiuc.edu/Research/vmd/; Humphrey et al., 1996) and rendered by Tachyon implemented in the VMD software package (Stone, 1998).

### Docking

Ligand structures were downloaded from https://pubchem.ncbi.nlm.nih.gov/ available and geometrically optimized by 500 steps of steepest descent under MMFF94 force field parameters included in Avogadro. Protein structures were prepared by Dock Prep, which is part of the Chimera software environment. The AMBER force field (AMBERff14SB) was applied to the receptor while Gasteiger charges were added to the ligand and co-factors. As recently reported, docking can be improved by assigning partial charges to metal ions (Hu and Shelver, 2003). In this context, Mg-ion charges were set to +1. Syn-CDP charge was set to −3. Docking was performed by AutoDock Vina using standard parameters. Docking poses were chosen based on a structural comparison to the pyrophosphate group that is co-crystallized in pdb 5A0J (see Figure S1B). The chosen pose was furthermore validated by re-dock approaches. Therefore, the predicted syn-CDP pose was *de novo* geometrically optimized by 500 steps of steepest descent under MMFF94 force field parameters included in Avogadro software environment prior to docking repetition (see Figure S1A).

### Model generation

An initial homology model of the ACS α-domain was predicted by RaptorX starting from the amino acid 565. A model based on the pdb crystal structure 5A0J, referring to a labdane related diterpene synthase, was manually selected for further structure function analyses. In order to prepare the model for docking studies, the coordinating Mg^2+^-ion triad and water molecules were implemented in the structure by different methods. Model A was generated by structural alignment to 5A0J. Cofactor positions were transferred from the structure template to Model A without any further adjustment prior to docking studies. Model B was created by MODELLER implemented in the Chimera software environment using the 5A0J as template structure. In this model hetero atoms and water molecules in the structure environment were computationally implemented. The pyrophosphate group was removed prior to docking with syn-CDP. Model C was prepared analogously to Model B but prior to refinement by MODELLER, syn-CDP was docked into the template structure 5A0J.

### Model validation

The protein ligand complex of Model B was validated by molecular dynamics studies. Therefore, syn-CDP was initially extracted from Model B and parameterized by CHARMM General Force Field program version 1.0.0 for use with CGenFF version 3.0.1. VMD was used to parameterize the protein and for merging ligand and protein. Subsequently, a water sphere was added around the protein-ligand complex. Two nanoseconds of molecular dynamic studies under CHARMM General Force Field was applied to the protein complex by NAMD. The calculated rmsd of the generated frames was plotted over time (Figure S2). A constant rmsd value was chosen as the criteria for an equilibrated protein-ligand complex. The last frame obtained was compared to the initial model B (Figure S3).

#### Plasmids for diterpene production

For all cloning procedures *E. coli* HMS 174 (DE3) was used. Clones were cultivated at 37°C in Luria-Bertani (LB) medium. Chloramphenicol (34 μg/L) and Kanamycin (50 μg/L) were added as required. For efficient production of the diterpene AD, *E. coli's* internal 1-deoxy-xylulose-5 phosphate pathway flux was increased by overexpression of deoxy-xylulose 5 phosphate synthase (dxs: GenBank: YP001461602.1), isopentenyl-diphosphate delta isomerase (idi: GenBank: AAC32208.1), and further extended by expressing geranylgeranyl diphosphate synthase (crtE: GenBank: KPA04564.1) and Aphidicolan-16-ß-ol synthase (acs: GenBank: AB049075.1). Therefore, dxs and acs were amplified from original sources by PCR. Polycistronic operons (Table 1) were constructed by BioBrick cloning standard (Shetty et al., 2008).

**Table 1:** Plasmids used for AD production in *E. coli*.

**Name**	**Promotor strength**	**Genes**	**Resistance**	**vector**
pAX dic	Weak	dxs, idi, crtE	Kanamycin	pBR322
pACYC acs	Strong	acs	Chloramphenicol	pACYC duet

Site directed mutations of acs were generated by PCR. Forward primers were designed exhibiting the respective mutation at the 5′ end while the corresponding reverse primers were phosphorylated at 5′ end (Table S1). PCR products were ligated by T4 Ligase prior to transformation. All amino acid exchanges were confirmed by sequencing.

### Production of diterpenes

All diterpene production experiments were performed in *E. coli* BL 21 (DE3). To investigate the product outcome of ACS mutants, pACYC acs plasmids were co-transformed with pAX dic. Cultivation was performed in minimal media supplemented with 6 g/L yeast extract and 30 g/L glycerol at 25°C. After 60 h the culture was extracted with a mixture of hexane, ethanol and ethyl acetate (1:1:1) (v/v/v) for 1 h. The extract was centrifuged at 10,000 g for 2 min. The upper, organic phase was directly analyzed for diterpene products via GC-MS.

### Diterpene analytics

GC-MS analyses of diterpenes was performed by a Trace GC Ultra with DSQII (Thermo Fisher Scientific). Therefore, 1 μL sample was loaded (Split 1/10) by TriPlus AS onto a SGE BPX5 column (30 m, I.D 0.25 mm, Film 0.25 μm). The initial column temperature was set to 160°C and maintained for 5 min before a temperature gradient at 8°C/min up to 320°C was applied. The final temperature was kept for additional 3 min. MS data were recorded at 70 eV (EI) and m/z (rel. intensity in %) as total ion current (TIC). The recorded m/z range was in between 50 to 650.

NMR spectra were recorded in CDCl_3_ with an Avance III 500 MHz (Bruker) at 300 K. ^1^H NMR chemical shifts are given in ppm relative to CDCl_3_ (δ = 7.26 ppm). The 2D experiments (HSQC) were performed using standard Bruker pulse sequences and parameters.

## Results and discussion

### Homology model refinement

**Figure 3:**
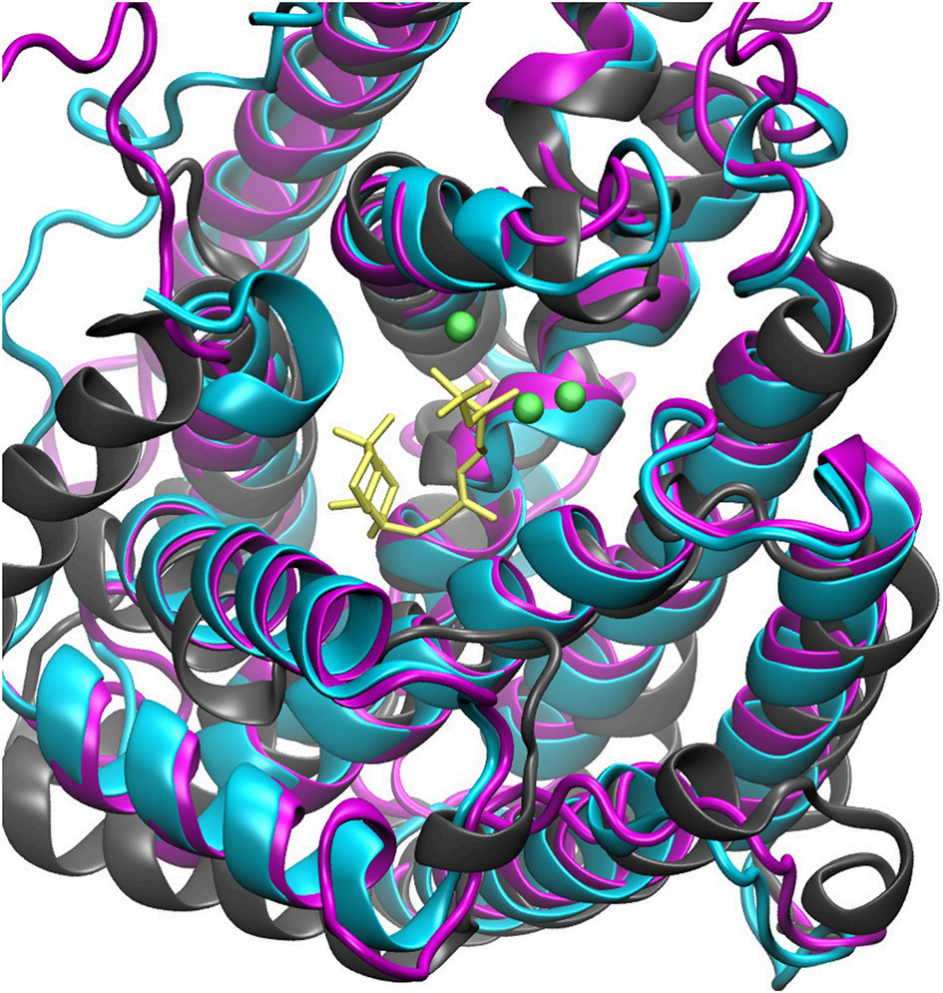
Structural alignment of Abietadiene synthase (gray), LRS (blue), and ACS (purple) in complex with Mg^2+^-ions and syn-CDP.

The steady increase in published protein crystal structures provides for an accelerated improvement of computational homology prediction. Especially due to the high structurally conservation of the terpene synthase enzyme families, biomolecular tools can predict structures solely based on the amino acid sequence. In this context, structure prediction of the bifunctional ACS was performed to analyze the highly complex conversion of GGDP via syn-CDP to the tetracyclic AD which is the core structure of the cytostatic compound Aphidicolin. ACS belongs to the diterpene synthase family and we identified three highly structurally conserved domains. The initial conversion from the universal diterpene precursor GGDP to syn-CDP occurs in class II active site, located between the ACS ß- and γ-domain. The subsequent syn-CDP cyclization to AD is then conducted in the class I active site that is positioned in the middle of an α-helical bundle forming the ACS α-domain. Notably, the fungal ACS is structurally highly similar to the previously crystallized plant diterpene synthases Abietadiene (pdb: 3S9V) and Taxadiene synthase (pdb: 3P5R), respectively. Homology prediction based on the full ACS sequence took those two structures into account, but for both, crystals could only be achieved in N-terminal truncated forms. Furthermore, these crystal structures have only been solved in an open conformation that is catalytically inactive. In order to circumvent the consideration of these catalytically inactive templates, only the ACS α-domain sequence was used for homology prediction. A model based on the labdane related diterpene synthases (LRS) (pdb: 5A0J), which is provided in a catalytically active holo-complex (Serrano-Posada et al., 2015), was selected for ACS homology refinement. The structural superposition of Abietadiene (pdb: 3S9V), LRS (pdb: 5A0J), and the ACS model, as depicted in Figure 3, explicitly demonstrates that there is a better fit between the ACS model and the LRS crystal structure. While the structural fit between LRS and the ACS model is visually well apparent, we have not calculated an rmsd value qualifier as structural domains that do not constitute the active site region are highly variable.

**Figure 4:**
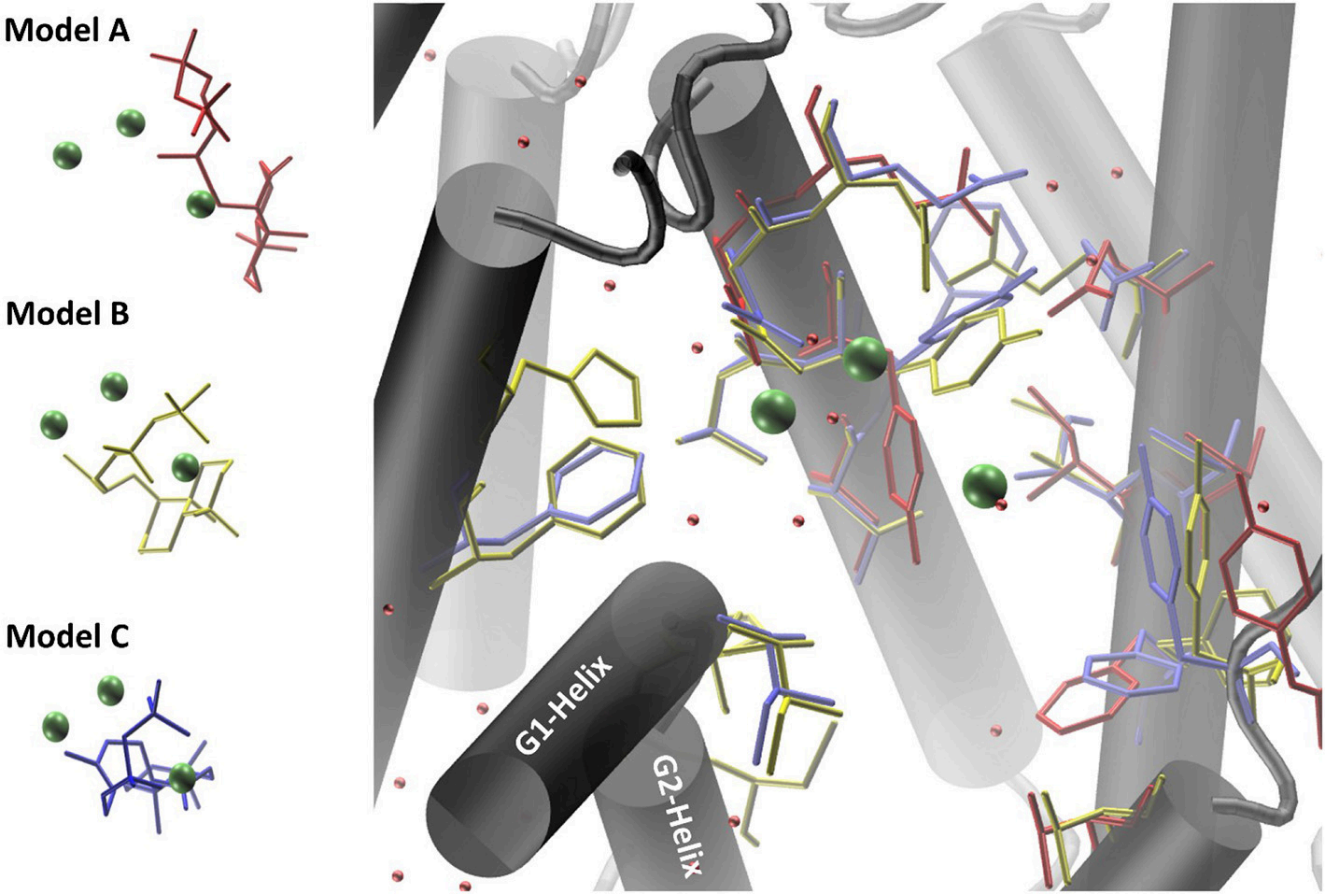
Homology models of ACS synthase refined and prepared for docking of the substrate syn-CDP. Model A results are colored red, Model B results yellow, and Model C results blue.

Co-crystallized cofactors (Mg^2+^-ion triad) and waters, both provided in the LRS structure, are also involved in the ACS reaction *en-route* from syn-CDP to AD. Therefore, we differentially adapted both, the positions of the Mg^2+^ ions and waters into the ACS models that resulted in the generation of three ACS models (A–C). Model A was prepared by adaptation of cofactor positions from the template structure LRS after structural alignment. Initial evaluation of this model indicated that this un-refined modeling method results in clashes of cofactors positions with amino acids side chains. Generally, in homology prediction the active site's cavity is not reserved for the substrate or cofactors specifically. Therefore, we presume that amino acid sidechains occupy this free space due to applied energy minimization optimizations. This is demonstrated in our docking studies of model A, where ACS amino acid Y658 is preventing syn-CDP to completely access the active site cavity. With the MODELLER package, which is based on comparative protein structure modeling by spatial restraints, a protein structure can be refined based on a template structure. Additionally, hetero-atoms and water molecules can be included directly in the model refinement. This refinement methodology applied to our initial model structure lead to the generation of ACS model B. This model B computationally included the three Mg^2+^-ions, a pyrophosphate group (conventionally derived by Mg^2+^ based hydrolysis of the phosphorylated substrate [syn-CDP] substrate) and water molecules directly as they are all present in the LRS template structure. Model B provides reliable positioning of the conserved amino acids that constitute the class I diterpene synthase signature DDXXD/E and NSE/DTE motifs in relation to the adapted Mg^2+^- ions, water and pyrophosphate moieties, respectively. Subsequently, we removed the pyrophosphate group from the model B structure to enable docking with the native syn-CDP substrate. Our docking data indicated that in Model B syn-CDP can completely access the active site's cavity. A specific syn-CDP conformation was selected pointing toward the ACS G-Helix, as this flexible helix is proposed to be involved in terpene cyclization reactions (Yoshikuni et al., 2006; Baer et al., 2014; Jia et al., 2017). This docking pose was validated by multiple re-docking approaches (Figure S1A). Additionally, we validated the pose while the position of the pyrophosphate moiety was compared to the pyrophosphate group co-crystallized in LRS (Figure S1B). Finally, a third approach for structure-function analyses was performed by docking syn-CDP into LRS prior to ACS refinement with MODELLER. Again, a syn-CDP conformation was chosen with close proximity toward the G-Helix. On the basis of this LRS holo-protein complex, an ACS holo-complex model C was generated. This method provided for a protein model that was refined around the substrate and cofactors. This methodology also provided for a precise specification of amino acids involved in the AD cyclization reaction. For all three models amino acids located within a five Ǻ vicinity to the docked substrate syn-CDP (thereby neglecting the pyrophosphate moiety) were analyzed by mutational studies to elucidate their catalytic relevance (see Figure 4, Table S2).

### Mutational validation of catalytically relevant ACS amino acids

**Figure 5:**
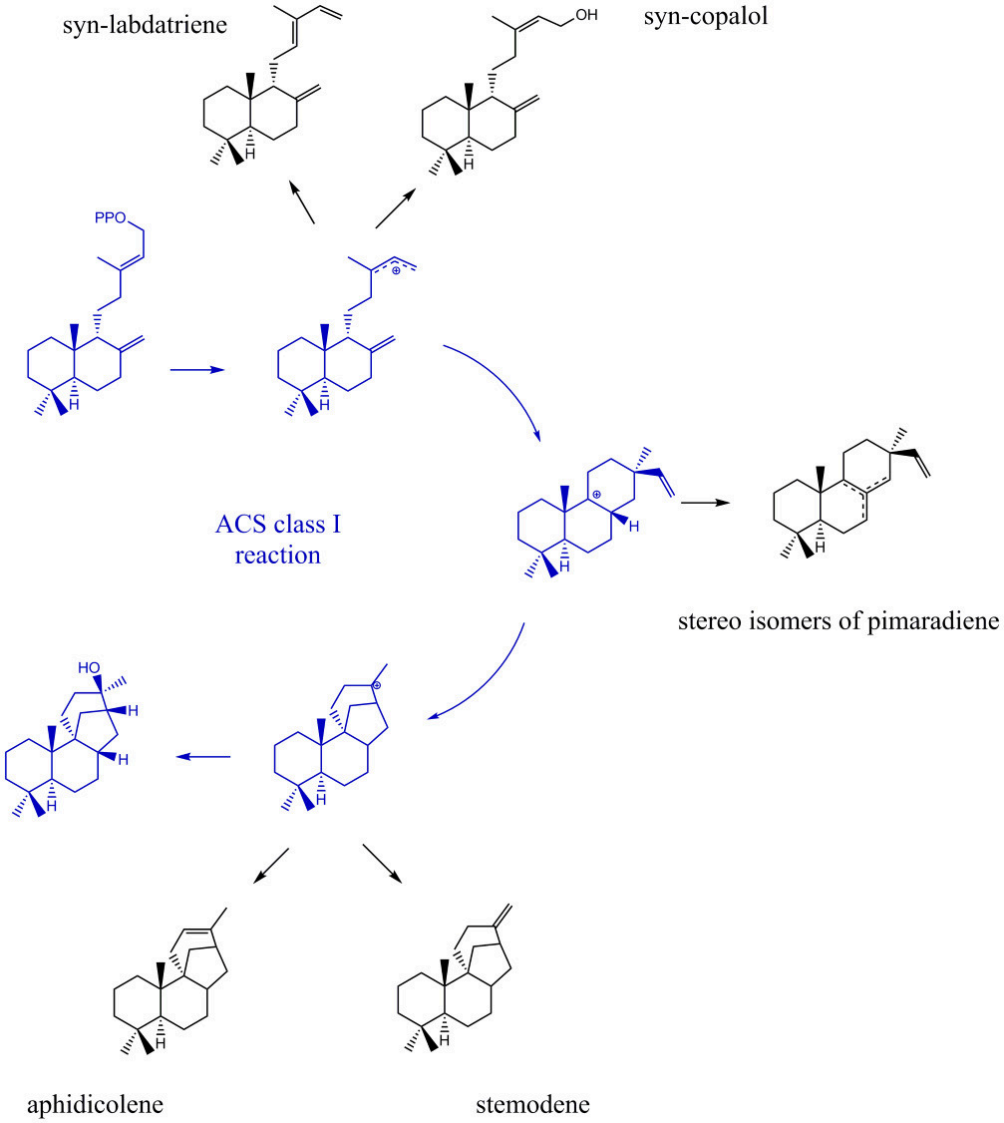
Expected products generated by ACS if the reaction cascade *en- route* from syn-CDP to AD is prematurely terminated.

Due to their stereo-chemical diversity, natural diterpene scaffolds are attractive research leads. The enormous stereo-chemical demand of diterpene macrocycles renders them difficult to access via total chemical synthesis approaches. Therefore, biosynthetic routes to generate these complex structures are currently an intense research focus (Dickschat, 2016; Bian et al., 2017; Jones, 2017). The ability to access new diterpene macrocycles via selective alteration of amino acids in diterpene synthases provides for a highly varied accessible chemical space. For the class I cyclooctat-9-en-7-ol synthase, which naturally generates a tricyclic fusicoccin type diterpene, amino acid mutations in the vicinity of the active site lead to intermittent abortion of the reaction cascade. Hence, alternative macrocyclic structures, such as the bicyclic dolabellane and the monocyclic cembrane, could be generated thereby elucidating the reaction cascade (Görner et al., 2013; Janke et al., 2014). In this study, insights into the class I reaction of the ACS were achieved by mutational studies. In that respect, we intended to quench the reaction from syn-CDP to AD at previously proposed transitional states (Adams and Bu'Lock, 1975; Oikawa et al., 2002). Based on the proposed ACS transitional states we presume that syn-labdatriene and syn-copalol (termination product of the syn-copalyl carbocation), stereoisomers of syn-pimaradiene (termination products of the pimaradienyl carbocation), or aphidicolene and stemodene (termination products of the aphidicolenyl carbocation) are potential abortion products (see Figure 5).

For an intermittent abortion of the reaction cascade from syn-CDP to AD, we have selected amino acids within a range of five Ǻ to the docked ligands as prime targets for mutagenesis (see Figure 4). Preliminary studies revealed that sidechain substitutions encompassing amino acids exchanges that inherently change physico-chemical properties frequently resulted in inactive enzyme variants (Janke et al., 2014; Schrepfer et al., 2016). In this context, we focused on changing the size of the respective amino acid sidechain thereby trying to preserve physico-chemical characteristics. Alternatively, we chose amino acid side chain substitutions that would replace polar groups with similar size amino acids (Table S2).

ACS syn-CDP docking results pointed toward a strong interaction between the decalin core and surrounding hydrophobic sidechains. However, as the decalin structure of syn-CDP remains untouched in further cyclization steps most of the implemented mutations near this particular moiety resulted in inactive (I626A, Y923L, F789L) or wildtype activity variants (F629L, Y658F, C831G, C831T, T920G, Y923F). Based on our modeling results, we also identified specific amino acids located in the ACS G-helix that in other studies have been proposed to be of catalytic relevance (Baer et al., 2014; Jia et al., 2017). While mutational changes in the G-Helix of Kaurene synthase like diterpene synthases resulted in alternative product profiles (Jia et al., 2017), our analogous approaches with ACS only provided inactive (A786L, F789L) or wildtype active (A786G, F789Y) variants. Nevertheless, our results support previous findings that propose the G-Helix as an essential flexible motif which is involved in the catalytically relevant structural change from the open to the closed enzyme configuration (Baer et al., 2014).

**Figure 6:**
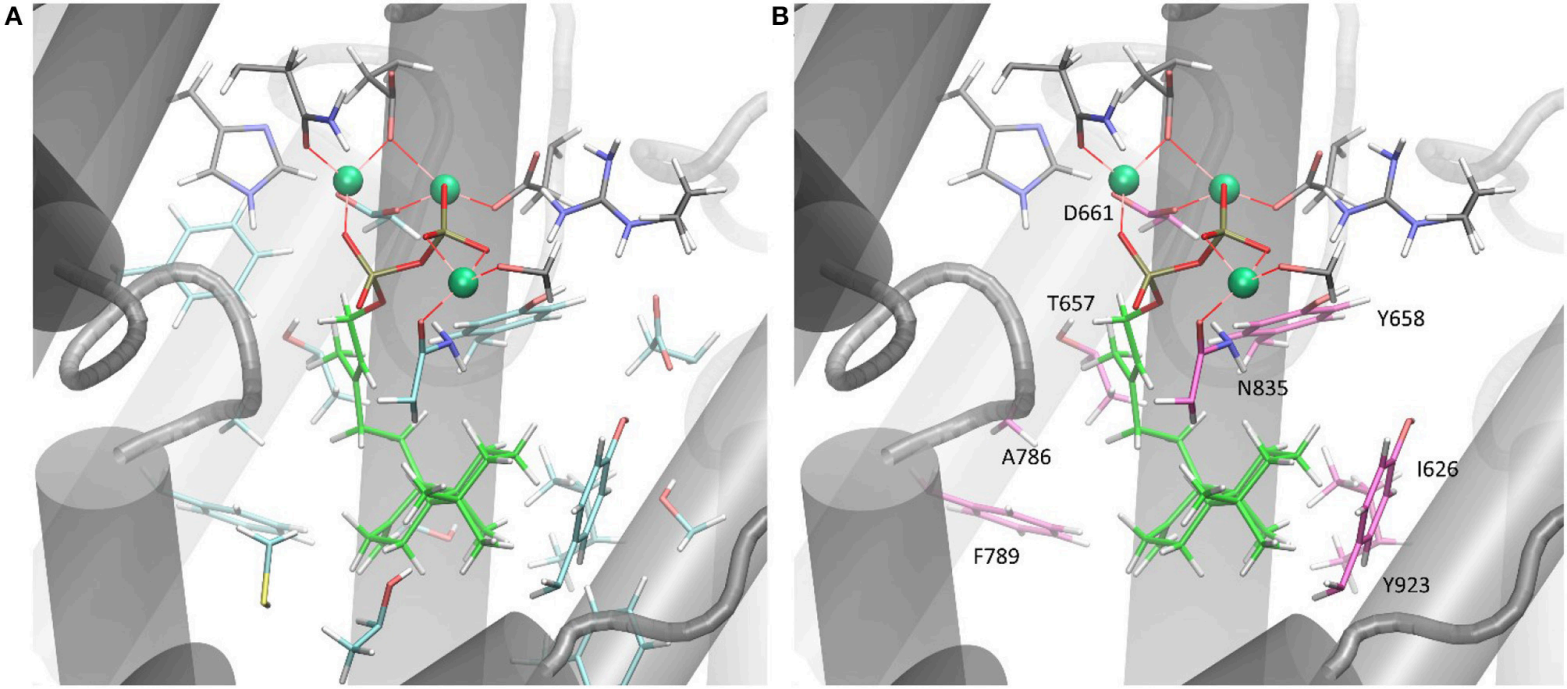
**(A)** ACS active sites cleft in complex with Mg^2+^ and syn-CDP. Amino acid network within five Ǻ to syn-CDP are displayed. **(B)** ACS active sites cleft in complex with Mg^2+^ and syn-CDP. Substitution of labeled amino acid (displayed in pink) resulted in inactive enzyme versions or mutants with altered product outcome.

Only the substitution of ACS Y658L and D661A provided for a varied product outcome. In addition to amino acids that constitute the DXXDD/E and NSE/DTE signature motifs that are responsible for Mg^2+^-ion coordination, our combined *in silico* and experimental study identified only a few amino acids (see Figure 6, colored in pink) capable to terminate activity. Our successful mutations (D661A, Y658L) indicated that the unusual cyclization from syn-CDP to AD proceeds in a spatially restricted area of the active site's cleft. Additionally, our data suggests that the pyrophosphate group remains in the active site and coordinates the reaction cascade. This is in accordance to the recently postulated Taxadiene synthase reaction mechanism (Schrepfer et al., 2016).

### ACS mutants D661A and Y658L

**Figure 7:**
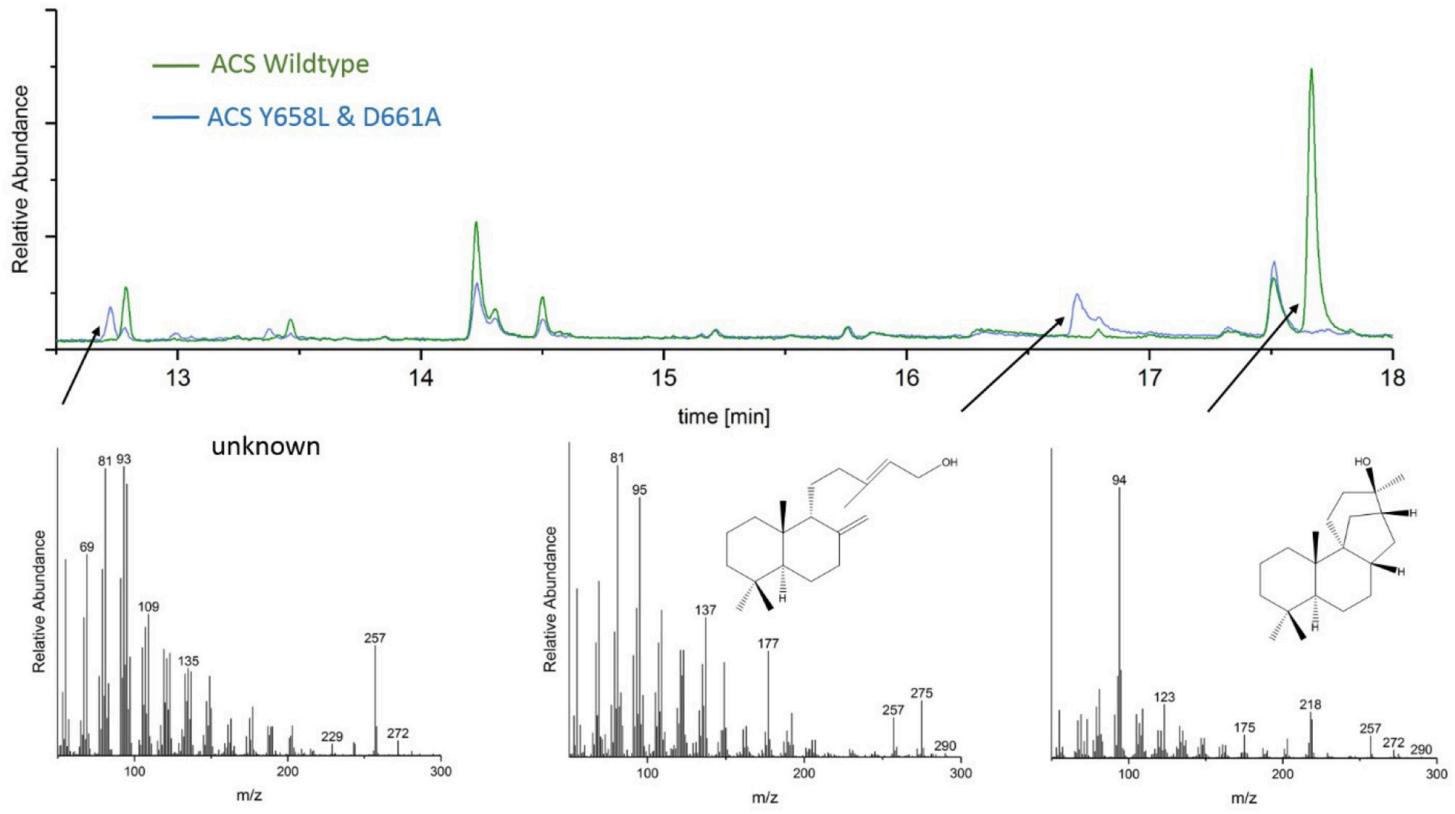
Analysis of ACS wildtype and ACS D661A mutant product outcome by GC. The MS-patterns for syn-labdatriene, syn-copalol and AD (from right to left) are presented below.

GC-MS analyses of the ACS mutants Y658L and D661A revealed that this mutations lead to the formation of two unknown diterpene products (see Figure 7). In contrast to the native AD, which had a GC retention time of 17.67 min, these new diterpenes had a retention time of 12.79 and 13.46 min, respectively. The latter product with a retention time of 13.46 min, showed a total mass of 290 m/z. Comparison of the MS spectral data suggests that this was a hydroxylated diterpene with a similar structure to syn-copalol (Hoshino et al., 2011). Subsequently, this compound was isolated and structural characterized by NMR (Figures S4, S5). The results are in accordance to previous spectral data for syn-copalol (Yee and Coates, 1992). One plausible explanation for syn-copalol formation is the quenching of the syn-copalyl carbocation intermediate by water in the active site of the enzyme. The other diterpene product with a retention time of 12.79 min had a total mass of 272 m/z indicating that this structure was not-hydroxylated. While we expected the formation of syn-labda-8(17),12E,14-triene, comparison with published MS-spectra revealed significant differences (Morrone et al., 2011). Unfortunately, due to the low amounts produced and purification issues for this highly hydrophobic compound, we could not conduct NMR analysis. However, we presume that this compound is also originated from the syn-copalyl carbocation and that a labdane related diterpene with high structural similarity to syn-labda-8(17),12E,14-triene was generated by the ACS mutants. The newly generated diterpenes are of great interest as copalol derivatives display various biological activities analogous to aphidicolin (Hanson, 2015).

The structural changes (D661A and Y658L) still allowed syn-CDP binding in the active site with subsequent hydrolyses of the pyrophosphate group. The syn-copalyl carbocation was then quenched either by water (release of syn-copalol) or an amino acid side chain (release of non-hydroxylated diterpene). Furthermore, as we did not find other substitution that stopped cyclization at the proposed transitional states and as we could not even detect changes in the byproduct formation of the active mutants, we presume that the ACS cyclization occurs in a spatially restricted area and that the pyrophosphate group remains in the active site, which is in accordance to recent reports (Schrepfer et al., 2016).

Former diterpene centered production processes were limited by low target compound yields. However, optimization of recombinant diterpene production hosts has extensively progressed to provide gram per liter yields (Ajikumar et al., 2010; Schalk et al., 2012). Today, access to novel diterpene lead structures is limited by the effective identification of relevant enzyme systems from large scale genome sequencing projects. Therefore, rational alteration of known terpene synthase product profiles by using a combination of *in silico* prediction and knowledge based mutagenesis studies can allow for a more rapid and targeted expansion of the desired chemical space.

## Conclusion

A model of ACS synthase was computed that required the application of various methods for model refinement to improve the quality of *in silico* structure function analysis. A model of the catalytically active, closed ACS α-domain complex was generated. Examination of this model provided for the identification of catalytically active amino acid sidechains. The *in silico* results were confirmed by mutational studies of the ACS. The amino acid substitutions Y658L and D661A in the vicinity of the ACS active site lead to formation of the alternative cyclization products syn-copalol and a minor labdane related diterpene. Formation of these products were delineated by quenching of the syn-copalyl carbocation *en-route* to AD. Additional mutants leading to inactive enzyme variants (A786L, F789L) provided insights into catalytically relevant amino acid residues within the G-Helix. The cumulative *in-silico* and experimental data suggests that amino acids constituting the G-loop motif of class I terpene cyclases are involved in the transformation of the open to the closed, catalytically active enzyme conformation. Moreover, as we only obtained a limited number of alternative cyclization products in our mutational screens, we presume that AD formation occurs in a rather confined location of the ACS active site. With respect to our biomolecular modeling approaches, we demonstrated that application of simple and rapid computational methodologies can be employed for prediction and structure function analyses of class I diterpene synthases.

## Author contributions

TB and MF supervised this study. MH initiated this study and performed virtual modeling and docking studies. NM and MM conducted mutagenesis experiments and screening under supervision of MH and MF. Data was analyzed by MH, MF, NM, MM, and TB. All figures were created by MH. All authors verified the data, contributed to the manuscript, and approved the final version.

### Conflict of interest statement

The authors declare that the research was conducted in the absence of any commercial or financial relationships that could be construed as a potential conflict of interest.

## Abbreviations

ACS: Aphidicolan-16-β-ol synthase
AD: Aphidicolan-16-β-ol
GGDP: geranylgeranyl diphosphate
LRS: labdane related diterpene synthase
syn-CDP: syn-copalyl diphosphate.
