# Supplementary material for: Insights Into the Bifunctional Aphidicolan-16-ß-ol Synthase Through Rapid Biomolecular Modeling Approaches
Source: Front Chem. 2018 Apr 10;6:101. doi: 10.3389/fchem.2018.00101 (PMC5902962; doi:10.3389/fchem.2018.00101)
Supplement: Supplementary file 1 [file DataSheet1.pdf]

Insights Into the Bifunctional Aphidicolan-16-ß-ol Synthase Through Rapid Biomolecular Modeling Approaches

M. Hirte[1], **N. Meese**[1], **M. Mertz**[1], **M. Fuchs**[1*], **T. Brück** [1*]

[1]Werner Siemens Chair of Synthetic Biotechnology, Department of Chemistry, Technical University of Munich, Germany

*** Correspondence:**
Corresponding Authors
Monika.fuchs@tum.de, brueck@tum.de

1 Supplementary Data

Amino acid sequence Aphidicolan-16-β-ol synthase:

MHPISTRSEVQDGLIAQARSLITRIVHNSDDVYGFGTLSCTVYDTAWVALVTKHVNGIKHWL
FPESFHYILASQCDDGTWCEDKTAQFDGVLNTIAGLLVLKRYRRDPLQLKVDNRDLDSRIKL
ATSALHSLLEEWDVSTTNNVGFEIIVPTMLDLLVQEDPSLSFELKGREALTEIREAKMNRFQP
ELLYQGKLMTLTHSLEALLGRIDYDNVARYTVKGSMFASPSSTAAYLMSASTWDDEAETYL
RYTITASTGKGSGGVPGVFPTTYFEYTWILSTLFRAGFQSSELDSPELTAMTDTLLKAFKAFS
GAIGLDSGIEPDVDDSAKIVTTLNMLGKPAHARYLCDNFEVETHFRVYPRERDPSFSANCNA
LAAFLHQPDVEVYSSQILKAASYLCERMWNADEKIDDKWNKSHLYSSFLYTQVMTDLMAI
TEAGRLDGVFTRELLTRVCVTIFQSCLRAMLKQSHDGSWNQSLEETAYAILHLTEARRLCFF
EQISEPLENSIYRGITFLTTIDKPPMEYLWSDKVSYGSAYLAETYVLAARRAAESTSVTNLVG
SSIWKDNASTKMHKLVGLFHRTPLLKALPKWELQASMIEASIYQGLLQDARLEVLQRPKVD
GGEYLSIIPFTWTSCSNSARTNASASHLWELMALSFFTYQVDEFMEAVAGPAFKGRMTHLH
AIIDEAVHCSQNRERTPGECENTNHVTSELLQAVRFILDNPTVRKASPYDRNTLLQELRIFLH
AHVTQVEDNASFGRERLTGDKALTSYRSQLYRWVHTISSDHIAGPFCFYYATCLLGATLTAH
PPNDCFPKSSQKYLAAATCRHLSCMCRMYNDIGSWNRDHREGNLNCLHFPEFSETTSDDAE
RKASLLTLAQYERKQWCNALQQLEKEMVHGASEPAAARLAKRRACLIDMFCKVTDLYGQI
YVLRDVSSVIKDVVRNGE*

Nucleic acid sequence Aphidicolan-16-β-ol synthase:

atgcatccaatttcgactcgttcagaagtccaggatggcttaatcgcccaagccagatccttgatcacacggatcgtccacaactccgacgacgttt
acgggtttggaacactaagttgcaccgtctatgatactgcttgggtggctttggttacgaaacatgtcaatggtatcaagcattggctgtttcctgaaa
gcttccattacattctcgcgtcacagtgcgacgatgggacttggtgcgaggacaaaacagcacagttcgacggagttctgaacacgatcgctgga
ctgcttgtcctaaaacggtacaggagagaccctcttcaactaaaagttgacaacagagatctcgattctcgtatcaaactcgcaacctctgcgctaca
ttccctgttggaagagtgggacgtttcgacaacgaacaatgttggctttgagatcatcgtcccaaccatgctggacctgttagtccaagaagatccat
cactctccttcgagctcaagggtcgtgaagcattgacagagatacgggaggctaagatgaatcgattccagccagaacttctataccaaggaaaat
tgatgaccttgacccactccttggaagcattgcttggcagaatagactacgacaatgttgctcgatatacagttaaaggctccatgtttgcgtcacctt
catccacggcggcctatttgatgagtgcgtctacatgggatgacgaggccgagacttacctacggtacaccatcactgcatcgacggggaaggg
aagcggtggtgttcctggcgtcttccctaccacatactttgagtacacatggattttgtctacactgtttcgagcgggattccaatcctcagaactcgac
tctcccgaactgaccgcaatgaccgatacactgctcaaagctttcaaggcattcagcggtgcgatcgggctagattccgggatcgagccagatgtt

gatgacagtgctaaaattgtcactacactcaacatgctgggtaagccggctcatgcacggtacttatgtgataactttgaggttgagacccattttcgc
gtatacccgagggaaagagacccaagctttagcgccaactgtaacgcgcttgctgcattcttacatcaacctgacgttgaggtgtactcatcacaga
tactcaaggcagcaagctatttatgtgagaggatgtggaacgcagacgagaagatcgacgacaaatggaacaagagccacctctattcaagcttc
ctctacactcaggtaatgacggacctgatggcaatcactgaggctggaagactagatggtgtgtttaccagagagctcttgaccagagtctgtgtga
ccatctttcaaagttgcctgagggcaatgttgaagcagtcacatgatgggtcatggaaccaatctttagaggagacagcctacgcgatcttgcatct
gacggaagcacgccggctctgcttctttgagcaaatttcggagccattggagaactccatctatcgtggaataacatttcttacaaccattgacaagc
caccaatggagtatctctggtctgacaaggtcagctacggctcagcctatctggcagagacatatgtcttagctgcccgaagggcagcagaatcca
cctcagtcaccaatcttgtcggttccagcatctggaaggacaacgcttcgacgaagatgcacaaactcgttggactttttcaccggacacccctcct
caaagcacttcctaaatgggagctccaagcttccatgatagaagcttccatctatcaaggccttctgcaggatgcacgattagaagtactgcagaga
cccaaggtagatggaggcgagtatctttccatcattcctttcacctggacaagctgtagcaacagtgctcgtacaaatgcttctgcatctcatttatgg
gagttgatggctttgtcattctttacatatcaggtggacgagttcatggaagccgtcgcaggaccagcattcaaaggacggatgacgcatcttcatgc
aatcatcgacgaagccgtacattgttcgcagaaccgggaaagaacaccaggagagtgtgaaaataccaatcacgtcacttccgagcttctgcaag
cagttcgcttcatactagacaaccctaccgtccgtaaagccagcccgtatgaccgcaacactttgttacaggagttacgaatcttccttcacgcacat
gtaacacaagttgaggataacgccagctttggaagagagaggttgacaggcgacaaggcccttacaagctatcgcagtcagctctaccgttgggt
gcacaccatatcatccgaccatattgccgggcctttttgcttctattatgcaacctgcttgttgggagctaccttgacagcacaccctccgaacgactg
ctttcccaagtcatcgcaaaagtatctggcagcagcgacatgtcgacacttgtcctgtatgtgtcgcatgtacaacgacatcggctcgtggaatcga
gatcaccgcgagggcaatctcaactgccttcacttccctgaattcagcgaaacgacctcggacgatgctgaaagaaaggcttctctacttactctcg
ctcagtacgagcgaaagcaatggtgcaatgcactacagcagcttgagaaagaaatggtacacggggcatcggagcctgctgcagcgaggctcg
cgaagcgtcgcgcatgtctgatagacatgttttgcaaagtgacggatctctatggacagatctatgttctccgcgatgtttcctccgtcatcaaggatg
ttgttcggaacggggaatag

2 Supplementary Tables

Table S1. Primers used for mutagenesis experiments

Name	Sequence
ACS A786 Rv-P	PHO-gatgatatggtgtgcaccc
ACS A786G Fw	cgaccatattggcgggcctttttgc
ACS A786L Fw	cgaccatattctggggcctttttgc
ACS C829 Rv-P	PHO-atacaggacaagtgtcgac
ACS C831G Fw	ggccgcatgtacaacgacatc
ACS C831T Fw	acccgcatgtacaacgacatc
ACS D661 Rv-P	PHO-cacctgatatgtaaagaatgac
ACS D661A Fw	gccgagttcatggaagccg
ACS E622A Fw	gcgtatctttccatcattcctttc
ACS E662 Rv-P	PHO-gcctccatctaccttgg
ACS F629 Rv-P	PHO-aggaatgatggaaagatactc
ACS F629L Fw	ctgacctggacaagctgtagc
ACS F741 Rv-P	PHO-gattcgtaactcctgtaac
ACS F741L Fw	ctgcttcacgcacatgtaac
ACS F789 Rv-P	PHO-aatatggtcggatgatatggtg
ACS F789L Fw	gccgggcctctgtgcttctattatg
ACS F789Y Fw	gccgggccttattgcttctattatg
ACS I626G Fw	tccggcattcctttcacctggac

ACS N835 Rv-P	PHO-gtacatgcgacacatacagg
ACS N835D Fw	gatgacatcggctcgtggaatc
ACS S625/ I626 Rv-P	PHO-aagatactcgcctccatc
ACS S625G Fw	ggcatcattcctttcacctgg
ACS S654G Fw	gggattctttacatatcaggtgg
ACS T657G Fw	gtcattctttggctatcaggtggacgag
ACS T657V Fw	gtcattctttgtgtatcaggtggacg
ACS T920 Rv-P	PHO-cactttgcaaaacatgtc
ACS T920G Fw	ggcgatctctatggacagatc
ACS Y658/S654/T657 Rv-P	PHO-aaagccatcaactcccataaatg
ACS Y658F Fw	gtcattctttacatttcaggtggacg
ACS Y658L Fw	gtcattctttacactgcaggtggacg
ACS Y923 Rv-P	PHO-gagatccgtcactttgc
ACS Y923F Fw	tttggacagatctatgttctccg
ACS Y923L Fw	cttggacagatctatgttctccg

Table S2. Experimental data of ACS mutants and wildtype for diterpene production

	Aphidicolan-16-β-ol	New diterpenes
**Wildtype**	+++	-
**E622A**	++	-
**S625G**	++	-
**I626A**	-	-
**F629L**	++	-
**S654G**	++	-
**T657G**	traces	-
**T657V**	+++	-
**Y658F**	+++	-
**Y658L**	-	++
**D661A**	-	++
**F741L**	+++	-
**A786G**	++	-
**A786L**	-	-
**F789L**	traces	-
**F789Y**	+++	-
**C831G**	++	-
**C831T**	+++	-
**N835D**	-	-
**T920G**	+++	-
**Y923F**	+++	-
**Y923L**	traces	-

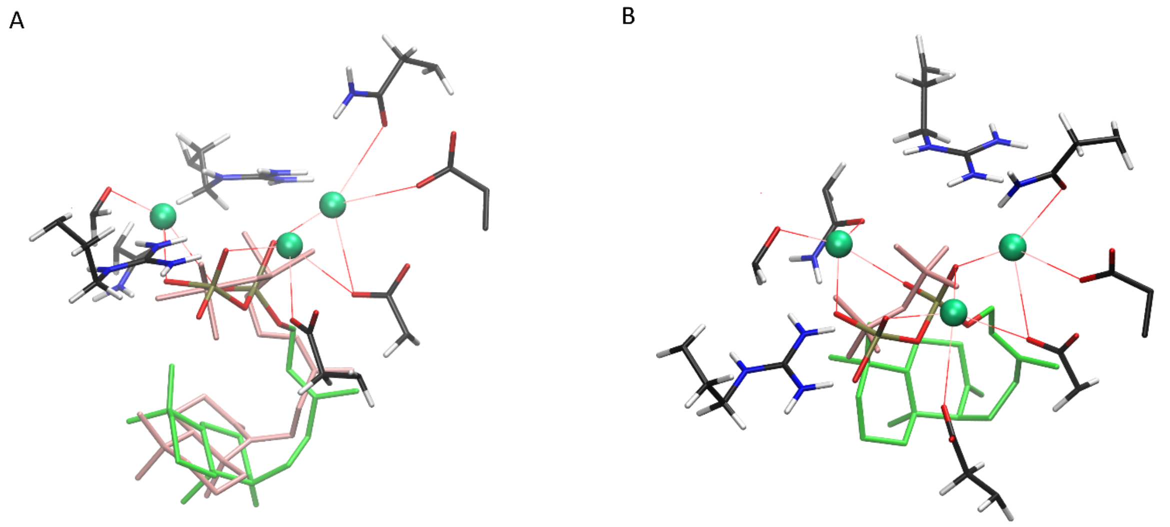

Figure S1. A Validation of docking results by re-docking geometrically optimization syn-CDP obtain from the initial docked ACS model (green: initial syn-CDP docking pose; pink: re-docked syn-CDP pose). **B** Validation of docking by superposition of the docked ACS-Model to pdb 5A0J and comparison of the co-crystallized pyrophosphate group (pink) to the docked syn-CDP (green).

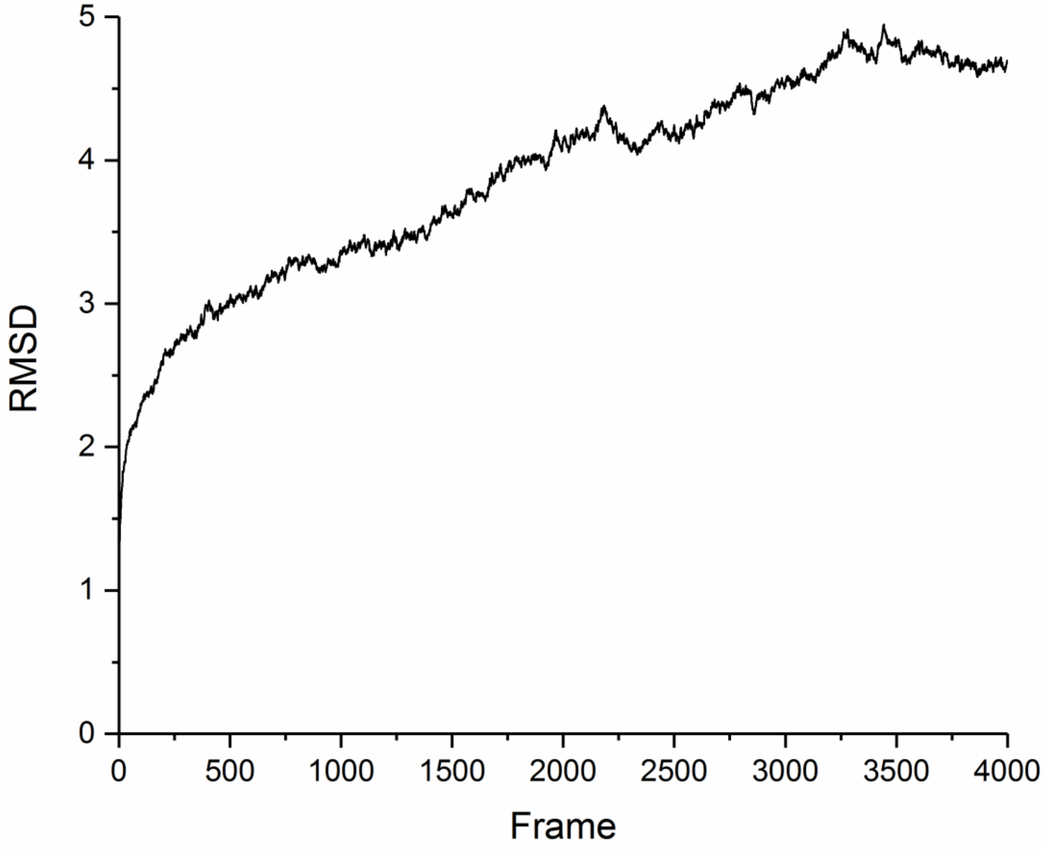

Figure S2. RMSD plotted against the ACS frames generated by 2 ns of molecular dynamic studies under CHARMM general force fields

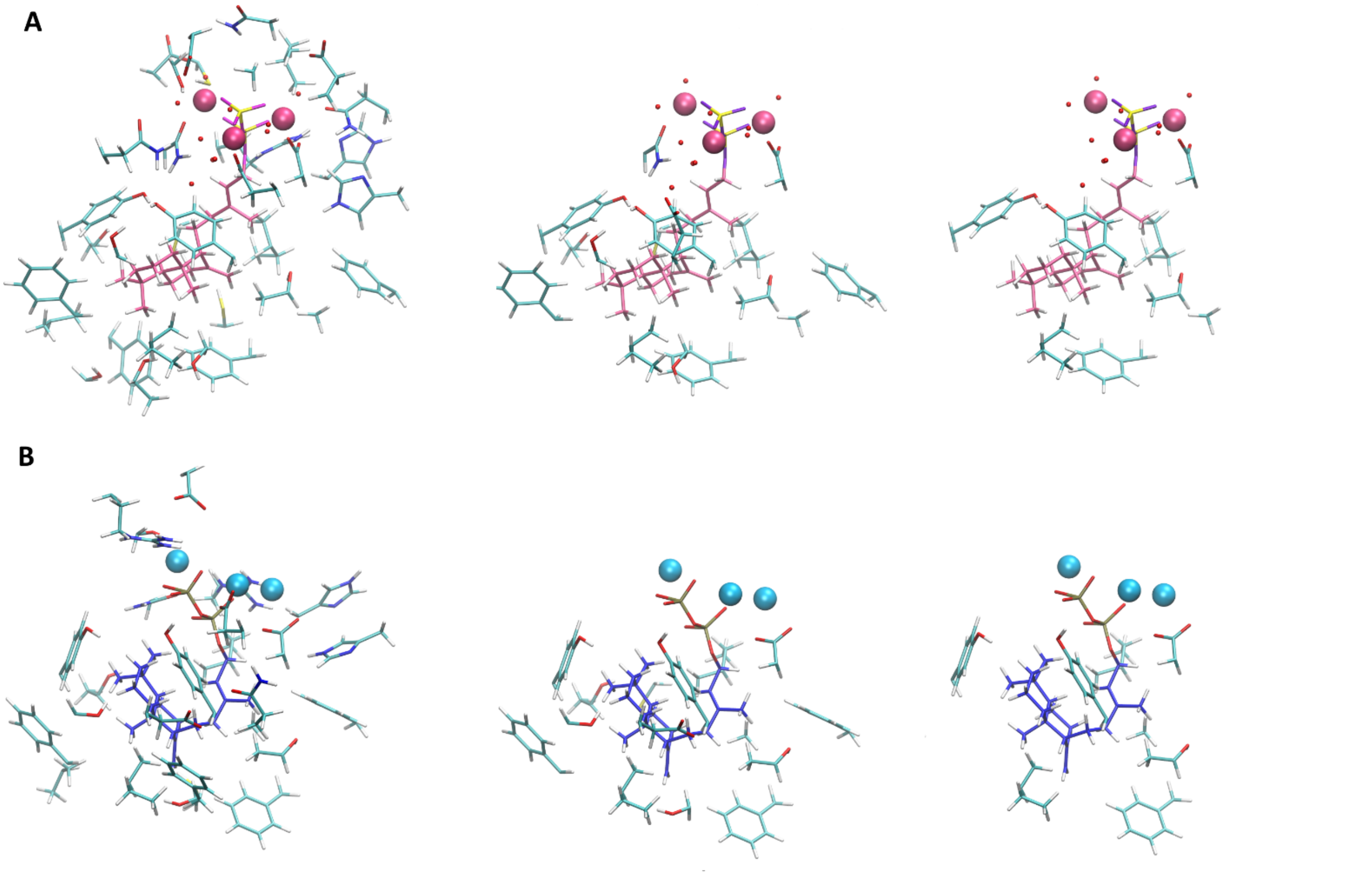

Figure S3. A: ACS model in complex with Mg^{2+} ions, water molecules and syn-CDP after 2 ns of molecular dynamic studies under CHARMM force field parameters. From left to right: Amino acid network within five Ǻ of syn-CDP; Screened amino acids; catalytic relevant amino acids. **B:** ACS model B in complex with Mg^{2+} ions and syn-CDP. From left to right: Amino acid network within five Ǻ of syn-CDP; Screened amino acids; catalytic relevant amino acids

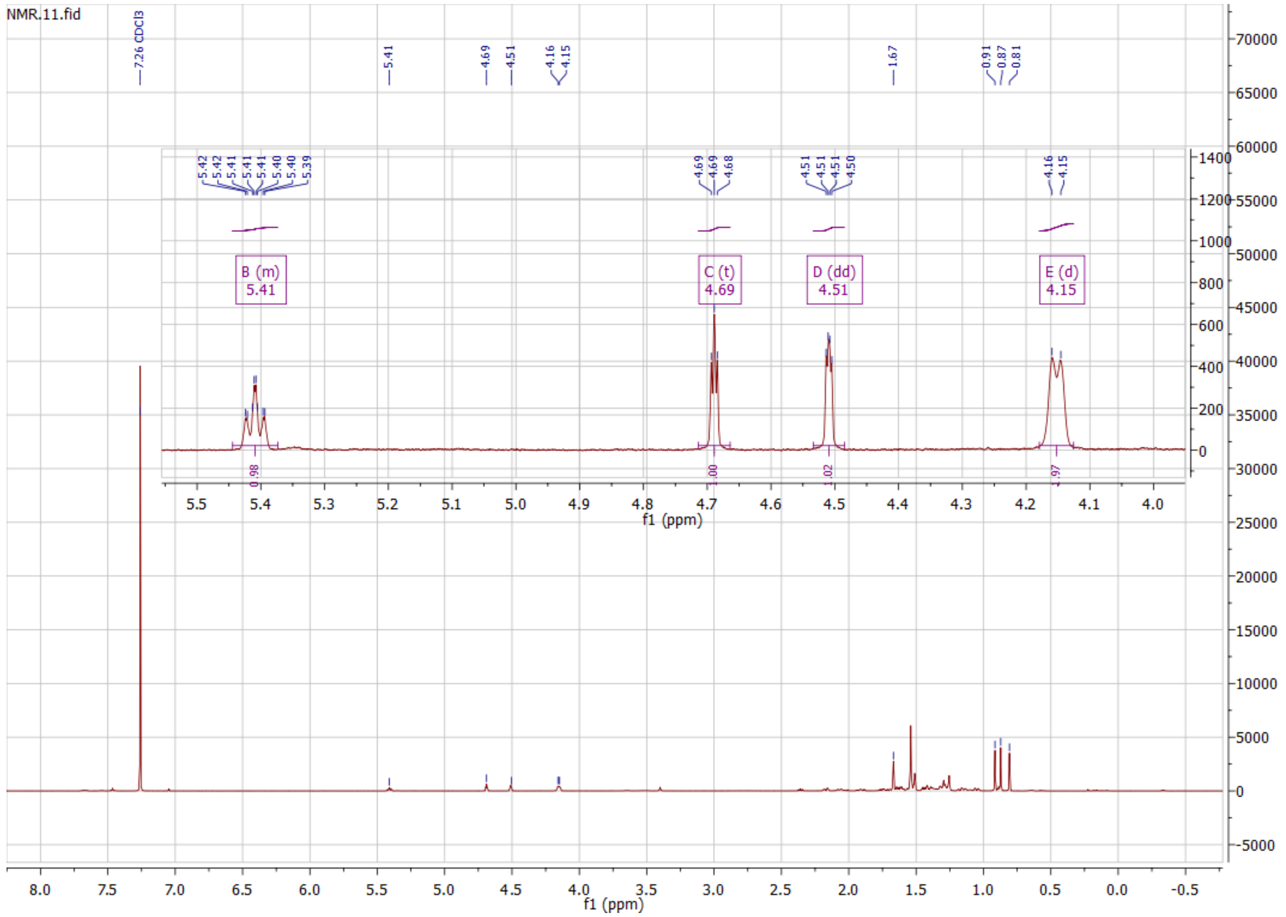

Figure S4. ^{1}H-NMR spectra of syn-copalol. Characteristic olefinic proton shifts of syn-copalol are labeled.

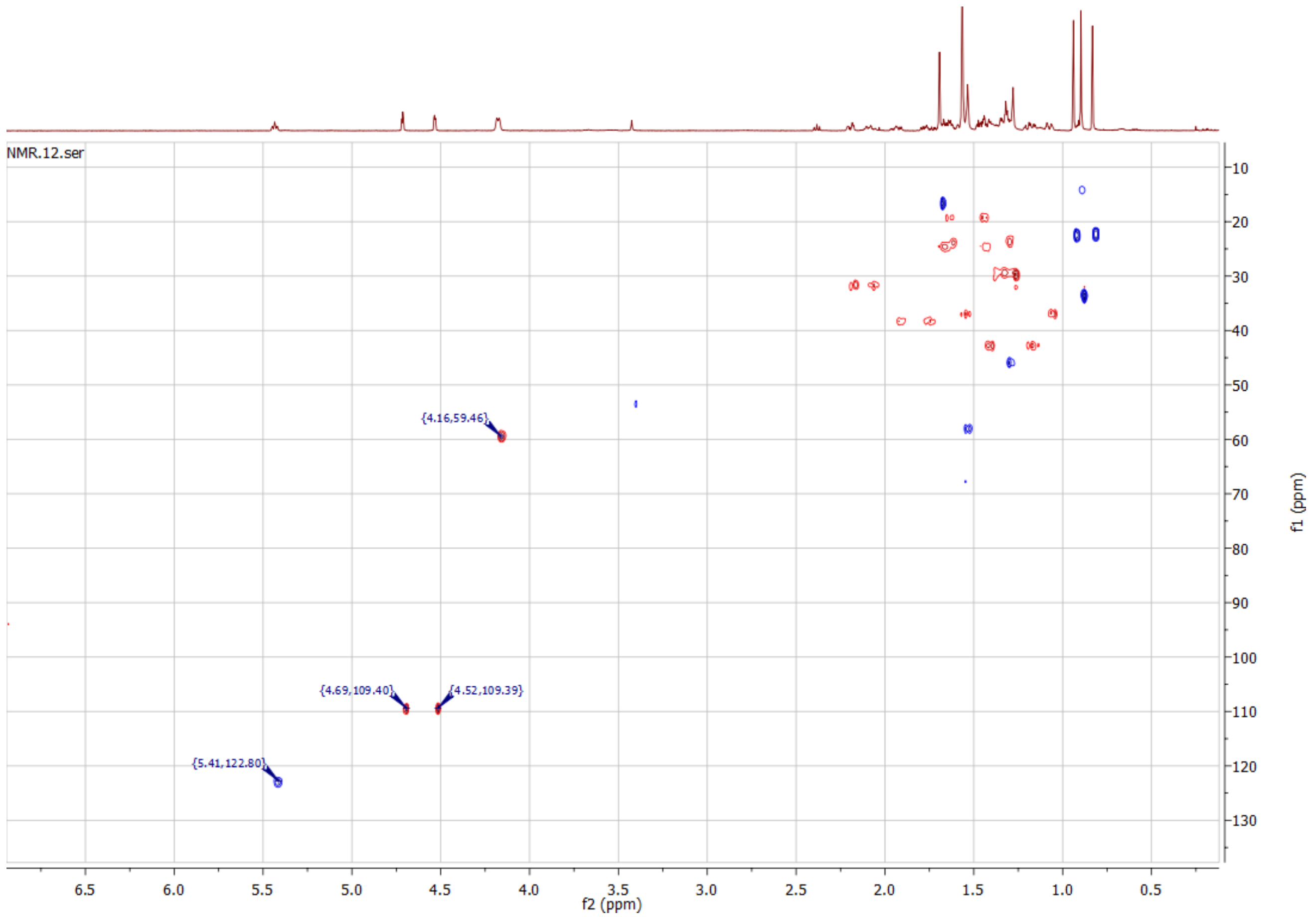

Figure S5. 2D NMR spectra of syn-copalol (HSQC). Characteristic olefinic protons that couple to their respective C-atom are labeled.
